# Circulating classical monocytes are associated with CD11c^+^ macrophages in human visceral adipose tissue

**DOI:** 10.1038/srep42665

**Published:** 2017-02-15

**Authors:** Kristiaan Wouters, Katrien Gaens, Mitchell Bijnen, Kenneth Verboven, Johan Jocken, Suzan Wetzels, Erwin Wijnands, Dominique Hansen, Marleen van Greevenbroek, Adriaan Duijvestijn, Erik A. L. Biessen, Ellen E. Blaak, Coen D. A. Stehouwer, Casper G. Schalkwijk

**Affiliations:** 1 Cardiovascular Research Institute Maastricht (CARIM), Maastricht University Medical Centre (MUMC), Maastricht, The Netherlands; 2 Department of Internal Medicine, MUMC, Maastricht, The Netherlands; 3 Department of Human Biology, NUTRIM School for Nutrition and Translational Research in Metabolism, MUMC, Maastricht, Netherlands; 4 Hasselt University, Biomedical Research Institute, REVAL-Rehabilitation Research Center, Diepenbeek, Belgium; 5 Department of Pathology, MUMC, Maastricht, The Netherlands; 6 Institute for Molecular Cardiovascular Research, RWTH Aachen, Germany

## Abstract

Immune cell accumulation in adipose tissue (AT) is associated with the development of AT inflammation, resulting in metabolic dysfunction. Circulating immune cell patterns may reflect immune cell accumulation in expanding AT. However, data linking human leukocytes in blood and AT is lacking. We investigated whether blood immune cell populations are associated with their counterparts in subcutaneous (scAT) or visceral AT (vAT). Flow cytometry was performed on blood, scAT and vAT from 16 lean and 29 obese men. Circulating natural killer (NK)-cells, classical monocytes and nonclassical monocytes were higher in obese individuals. vAT, but not scAT, of obese individuals contained more inflammatory CD11c^+^ “M1” macrophages and NK cells compared to lean individuals. Blood classical monocytes were associated with CD11c^+^ macrophages in vAT but not scAT. This association was unrelated to expression of the adhesion molecules CD11b and CD11c or of the chemokine receptor CX3CR1 on these monocytes. Other AT immune cells were not associated with their respective counterparts in blood. Finally, CD11c^+^ macrophages and CD4^+^ T-cells in vAT were associated with their counterparts in scAT. In conclusion, blood classical monocytes reflect CD11c^+^ macrophages in vAT.

Adipose tissue (AT) inflammation is a key feature of obesity-associated metabolic dysfunction^1^,^2^. Pro-inflammatory so-called “M1” macrophages are thought to be recruited from the circulation to AT^3^, where they accumulate around dying adipocytes to form crown-like structures (CLS), and contribute to insulin resistance and the risk to develop type 2 diabetes^4^,^5^. The presence of CD11c on the surface of human AT macrophages was shown to be discriminative for these CLS-macrophages and the presence of CD11c^+^ macrophages correlates with insulin resistance in humans^5^. Additionally, immune cells such as T-lymphocytes, dynamically change in AT during obesity, contributing to chronic inflammation^6^. Compared to subcutaneous AT (scAT), visceral AT (vAT) is thought to be the most inflammatory fat depot in obese people^7^. Unfortunately, vAT is not readily accessible for clinical assessment. Therefore, clinical research considers blood immune cells or scAT inflammatory status as a measure of AT inflammation. However, the validity of immune cells in blood or scAT as surrogate readout for the vAT immune cell profile is unknown.

Circulating immune cells, including monocytes, granulocytes and lymphocytes, are associated with obesity and its complications^8^. Monocytes, the precursor cells of macrophages, are subdivided based on surface expression of the lipopolysaccharide receptor CD14 and the Fc gamma receptor CD16 into CD14^+^ CD16^−^ (classical), CD14^+^ CD16^+^ (intermediate), and CD14^dim^CD16^+^ (non-classical) monocytes, representing 75–85%, 5–10% and 5–10% respectively of total blood monocytes^9^. Since at least part of AT-contained immune cells is recruited from the circulation, it seems plausible that blood immune cell patterns relate to inflammation in expanding vAT. Circulating CD16^+^ monocytes (including both intermediate and nonclassical) were shown to be associated with scAT total macrophage content as assessed by immunohistochemical staining of CD68^10^. However, data linking blood monocyte subtypes with specific macrophage subsets in vAT or scAT is unavailable, as is information on other leukocyte subsets^8^. Therefore, we investigated the cross-association of relevant leukocyte subsets in blood, scAT and vAT from lean and obese men using flow cytometry. Moreover, we investigated whether immune cells in accessible scAT reflect immune cell content in vAT.

## Research Design and Methods

### Subjects

Male subjects who were scheduled to undergo abdominal or bariatric surgery were recruited. The decision to undergo surgery was made independently of this research protocol, between patient and physician. The control group consisted of 16 lean male subjects. The obese group was composed of 29 male subjects. Within obese individuals there were 6 patients with type 2 diabetes (defined as having HbA1c >6.5% or taking glucose-lowering medication). 4 obese individuals were on glucose-lowering medication, one of which in combination with lipid lowering medication and 6 patients were on lipid-lowering medication only. Lean individuals were included due to surgery of inguinal hernia, except for 1 patient who underwent sigmoidectomy due to diverticulitis and 1 patient who underwent cholecystectomy due to cholesterolosis. Both patients showed some degree of inflammation but with respect to the circulating or AT immune cells, these patients fell within the normal range of variability.

Clinical characteristics of the subjects included in the study are summarized in Table 1. Major exclusion criteria were presence of a history of heart, lung or kidney disease and/or presence of endocrine anomalies. Lean controls were selected based on their age to obtain a group of which average age was not different from the obese group. The study protocol was approved by the Medical Ethical Committee Jessa hospital, Hasselt, and Hasselt University, Belgium, in accordance with the Declaration of Helsinki, and all subjects gave their written informed consent before participating in the study.

### Anthropometric measurements and blood sampling

Body weight, height, waist/hip circumference and blood pressure were determined at the morning of surgery. Fat and lean body mass were estimated by bio-electrical impedance analyses (Bodystat 1500; Bodystat Ltd., Isle of Man, U.K.). Fasting venous blood samples were collected after an overnight fast for measurement of plasma glucose, serum insulin and glycated hemoglobin. Plasma glucose was measured by the glucose oxidase method using an AU2700 analyser (Beckman Coulter, Brea, CA, USA). Serum insulin was assessed by an immunoassay (ADVIA Centaur Insulin IRI; Siemens Medical Solutions Diagnostics, Tarrytown, NY, USA). Glycated hemoglobin was assessed by high performance liquid chromatography using a HA-8160 Hi-Auto A1C analyser (Menarini, Zaventem, Belgium). Blood cell counts were done with ADVIA 2120 Hematology System (Siemens) and are expressed as cells/L.

### Adipose tissue biopsies

After an overnight fast, adipose tissue biopsies were taken from the abdominal subcutaneous adipose tissue depot (scAT) and the omentum majus (vAT). For each adipose tissue depot, approximately 1–3 g adipose tissue was collected. The tissue was immediately brought to the laboratory for further processing.

### Stromal vascular Fraction (SVF) preparation

SVF cells were obtained by collagenase digestion of adipose tissue fragments in Dulbecco’s Modified Eagle’s Medium (DMEM)-Ham’s F12 with Collagenase 11 (Sigma Aldrich C7657, 0.0875 mg/ml), collagenase 1 (Sigma Aldrich C0130, 1.216 mg/ml) and DNAse 1 (Sigma Aldrich DN25, 0.09 mg/ml) under gentle shaking (60 cycles/min) at 37 °C. The resulting suspension was filtered through a 200 μm filter and fat cells were removed as floating fraction after centrifugation (5 min, 1250 rpm). SVF cells were washed once with DMEM-Ham’s F12 to eliminate collagenase, filtered through a 70 μM filter and stained for flow cytometry.

### Flow cytometry

Isolated SVF cells were used for two antibody cocktails. Cocktail 1 included CD45-PE-Cy7 (BD 557748), CD3-fitc (BD 561807), CD19-fitc (BD 555412), CD56-fitc (BD 562794), CD66b-fitc (BD 555724), CD11b-BV421 (Biologend 301324), and CD11c-APC-Cy7 (Biolegend 337218). Cocktail 2 included CD45-PE-Cy7 (BD 557748), CD3-V500 (BD 561416), CD4-PerCP (Biolegend 300528), CD8 APC-H7 (BD 641400), CD19-BV421 (Biolegend 302234), and CD56-APC (Biolegend 318310). Samples were measured with a FACS-Canto II (BD Biosciences). Results were analysed with FACSdiva (BD Biosciences) and FlowJo software. Gating strategy is shown in supplemental Fig. 1. Since weight of the adipose tissues was unavailable, data are expressed as % of cells relative to total cells (based on forward and side scatter plot).

Detailed characterization of blood cells was achieved using flow cytometry. Heparinized whole blood was used for staining for flow cytometry using two antibody cocktails (50 μl of whole blood for each staining). Cocktail 1 included CD3-fitc (BD 561807), CD19-fitc (BD 555412), CD66b-fitc (BD 555724), CD56-PE (BD, 345810), HLA-DR-V500 (BD 561224), CD14-APC-H7 (BD 641394), CD16-PerCP (BD 560717), CD11b-BV421 (BD 562632), CD11c-PE-Cy7 (BD 561356), and CX3CR1-APC (Biolegend 341609). Cocktail 2 included CD3-V500 (BD 561416), CD4-APC-H7 (BD 560158), CD8-PerCP-Cy5.5 (BD 560662), CD25-PE-Cy7 (BD 335824), CD127-fitc (BD 560549) and CD19-PerCP-Cy5.5 (BD 332780). Since it was our goal to detect correlations between the different immune cell depots, data on circulating immune cells are expressed in the same manner as for tissue macrophages, i.e. as % of total cells. Notably, white blood cell counts did not differ between the groups.

Analyses were performed on fresh blood samples. To standardize measurements, we used antibodies from a single batch during the whole study. Upon arrival, antibody solutions were aliquoted into small volumes to avoid frequent temperature changes or contamination. Furthermore, cytometer tracking beads were used bi-weekly to define a baseline and standardize daily measurements of the cytometer.

### Statistical analyses

All analyses were performed with the Statistical Package for Social Sciences (SPSS) version 17.0 for Windows (SPSS Inc, Chicago, Illinois, USA). Depending on missing variables, data on n = 16 for the lean and between 21 and 29 for the obese individuals were included in analyses. Variables with a skewed distribution (based on Shapiro-Wilk test) were ln transformed prior to analysis. Comparison of baseline characteristics between lean and obese subjects was performed with an independent Student’s t-test. Additionally, differences in immune cell populations between lean and obese subjects were analysed with linear regression analyses and were adjusted for age. Linear regression analyses were also used to investigate the associations between circulating immune cell populations and immune cell populations in scAT and vAT.

## Results

### Immune cell profiles in blood, scAT and vAT of lean and obese men

Total circulating white blood cells, lymphocytes, monocytes, basophils, eosinophils and neutrophils did not differ between lean and obese individuals (Table 2). Multicolour flow cytometry showed that the percentage of blood nonclassical monocytes, classical monocytes, and NK-cells out of total leukocytes was higher in obese individuals compared to lean controls. No significant differences were found between lean and obese men for circulating B-cells or T-cell subsets (Table 2). vAT from obese individuals was enriched in CD45^+^ immune cells, CD11c^+^ macrophages and NK cells relative to total stromal vascular cells. The relative abundance of CD11c^−^ macrophages and B-cells tended to be higher in these subjects (Table 3). Interestingly, these differences were not observed in scAT, confirming that scAT is less prone to obesity-associated adipose inflammation than vAT^7^.

### Circulating classical monocytes are associated with CD11c^+^ macrophages in vAT, independent of surface integrin and CX3CR1 expression

**Figure 1:**
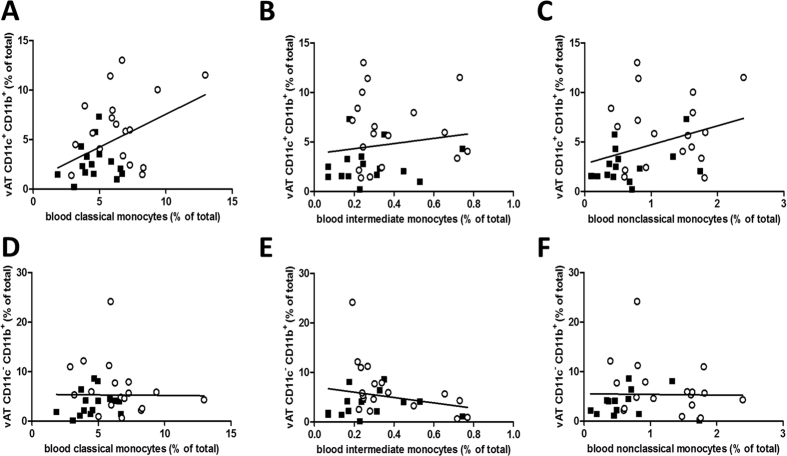
Leukocytes in vAT (represented as % of stromal vascular cells on the Y-axis) were associated with circulating monocyte subsets (represented as % of leukocytes on the X-axis) and with scAT leukocytes (represented as % of stromal vascular cells on the X-axis). Squares represent data of lean individuals and open circles represent data from obese individuals. Regression analyses are shown for CD11c^+^ CD11b^+^ M1 macrophages with (**A**) classical monocytes (β = 0.67, p = 0.014), (**B**) intermediate monocytes (β = 2.61, p = 0.40) and (**C**) nonclassical monocytes (β = 1.90, p = 0.054) and for CD11c^−^CD11b^+^ M2 macrophages in vAT with circulating (**D**) classical monocytes (β = −0.023, p = 0.95), (**E**) intermediate monocytes (β = −5.54, p = 0.20) and (**F**) nonclassical monocytes (β = 0.41, p = 0.77).

We investigated whether blood immune cell profiles relative to total leukocytes are associated with corresponding immune cells relative to stromal vascular cells in both AT depots. We found a positive association of blood classical monocytes with CD11c^+^ macrophages in vAT (β = 0.67, p = 0.014) (Fig. 1A). Neither intermediate (β = 2.61, p = 0.40) (Fig. 1B), nor nonclassical circulating monocytes (β = 1.9, p = 0.054) (Fig. 1C) correlated with CD11c^+^ macrophages in vAT. None of the circulating blood monocytes were associated with CD11c^−^ vAT macrophages (Fig. 1D–F). Analysing lean and obese groups separately no longer showed a significant association, probably due to lack of power. However, effect sizes remained similar. Other blood immune cell types were not associated with their corresponding population in vAT (B-cells: β = 0.008, p = 0.97; CD4^+^ T-cells: β = 0.136, p = 0.67; Th-cells: β = 0.149, p = 0.67; Treg-cells: β = 1.140, p = 0.68; CD8^+^ Tc-cells: β = 0.064, p = 0.82; NK-cells: β = 0.089, p = 0.50). In addition, blood monocyte subsets were not associated with either macrophage subset in scAT, nor were blood lymphocytes associated with their presence in scAT (data not shown). Excluding obese patients with type 2 diabetes from these analyses did not materially alter these results (data not shown).

Integrins are involved in trafficking^11^, adhesion and transmigration of monocytes^12^ and may be markers of cellular activation^13^. Therefore, we assessed whether surface integrin expression on blood monocytes, measured as the mean fluorescence intensity (MFI), was involved in the association found between blood monocyte and AT macrophage content. The expression of the integrin CD11b by classical monocytes was higher in obese individuals (Table 2). However, our analysis did not reveal any association between CD11b expression by monocyte subsets and CD11c^+^ macrophage accumulation in vAT (classical: β = 0.018, p = 0.81; intermediate: β = 0.045, p = 0.60; nonclassical: β = 0.084, p = 0.79). Similar null-results were obtained for vAT CD11c^−^ macrophages (data not shown). Expression of the integrin CD11c on circulating monocytes did not differ between obese *vs* lean individuals (Table 2). In addition, the fractalkine receptor CX3CR1 has been suggested to be involved in monocyte recruitment during metabolic inflammation^14^. CX3CR1 expression on classical monocytes was not associated with CD11c^+^ macrophage accumulation in vAT (β = −0.09, p = 0.80). CX3CR1 expression on intermediate monocytes however, did show a significant association with CD11c^+^ macrophage accumulation in vAT (β = 0.35; p = 0.039) and a similar trend was found for nonclassical monocytes (β = 0.31; p = 0.056). No associations were found for CX3CR1 expression with vAT CD11c^−^ macrophages (data not shown).

### vAT CD11c^+^ macrophage and Th-cell content is reflected by scAT

**Figure 2:**
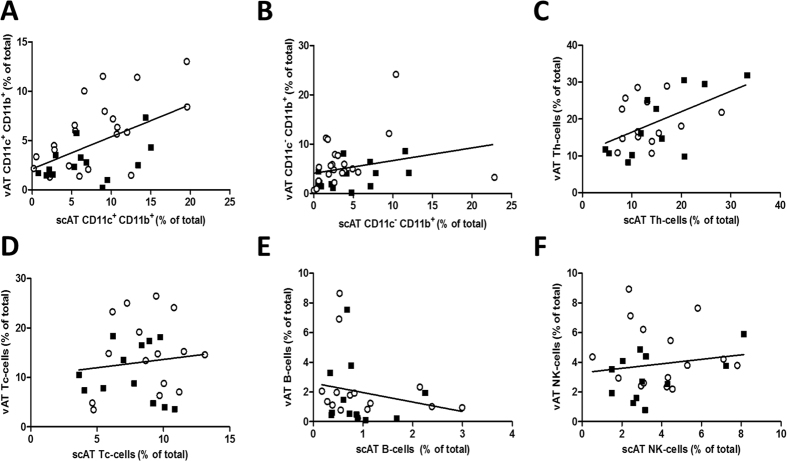
Regression analyses between scAT and vAT are shown for (**A**) CD11c^+^ CD11b^+^ M1 macrophages (β = 0.36, p < 0.001), (**B**) CD11c^−^CD11b^+^ M2 macrophages (β = 0.26, p = 0.12), (**C**) CD4^+^ Th cells (β = 0.61, p = 0.006), (**D**) CD8^+^ Tc cells (β = 0.33, p = 0.55), (**E**) B-cells (β = −0.64, p = 0.31), and (**F**) NK cells (β = 0.15, p = 0.45).

We investigate whether immune cells in accessible scAT reflect immune cell distribution in vAT by determining their association between vAT and scAT. A significant association was found for CD11c^+^ macrophages (β = 0.37; p < 0.001) (Fig. 2A), but not for the CD11c^−^ subset (β = 0.26; p = 0.12) (Fig. 2B). In addition, an association was found for CD4^+^ T-helper cells (β = 0.61; p = 0.006) (Fig. 2C), but not for other lymphocytes (CD8^+^ Tc-cells, B-cells, and NK cells) (Fig. 1D–F). Excluding obese patients with diabetes from these analyses did not materially alter these results (data not shown). Finally, we performed these analyses separately in lean and obese individuals. The results show that obese individuals contributed most to the association of CD11c^+^ macrophages between vAT and scAT (β = 0.41, p = 0.003); while lean individuals only retained a tendency (β = 0.18, p = 0.096). Remarkably, the association between scAT and vAT Th-cells depended on lean individuals (β = 0.79, p = 0.006); while this association was completely lost in the obese group (β = 0.31, p = 0.42).

## Discussion

Our data delineate the associations between immune cell populations in human blood, vAT and scAT.

The flow cytometry data in blood of lean and obese individuals are in agreement with numerous studies showing positive associations between circulating nonclassical monocytes and obesity^8^,^15^. In contrast, data on classical monocytes seem inconsistent as not all studies find these cells to be increased in obese individuals^7^,^10^. In line with our data however, Schipper *et al*. showed that classical monocytes associate with BMI in obese children^13^. It should be noted that many studies report data on monocyte subsets either relative to the total monocyte pool, or as cell number. For the comparison with AT immune cell subsets, we have expressed our data as percentage of cells relative to total leukocytes, similar to the representation in AT (as % of stromal vascular cells). Such different representation of data within the current literature may partially explain differences between studies.

Importantly, our analyses show that classical monocytes in blood reflect the accumulation of inflammatory CD11c^+^ macrophages in vAT. These correlations may indicate that the accumulation of vAT CD11c^+^ macrophages originates at least in part from blood monocytes. This observation seems at odds with recent cell tracking studies in mouse obesity models which show that, at least in the steady state, macrophages are seeded in AT during embryogenesis and are maintained by local self-renewal, independent of monocyte recruitment (15). Interestingly, Haase *et al*. reported that local proliferation contributes mainly to M2 macrophages in murine adipose tissue^16^. In addition, CD68^+^ macrophages in human obese compared to lean AT showed increased expression of proliferation markers, but it remained unclear whether this was associated with a specific macrophage subset^16^. Our data corroborate the concept that CD11c^+^ “M1” macrophages in human vAT originate from circulating classical monocytes. In line, Pecht *et al*. recently showed that classical monocytes from healthy male donors displayed the highest migration toward conditioned medium from vAT compared to nonclassical and intermediate monocytes from the same donors^17^. Clearly, it is complicated to resolve these issues in humans. In mice, a recent elegant study using shielded irradiation followed by bone marrow transplantation showed that during obesity, monocyte influx and monocyte-to-macrophage differentiation in AT becomes increasingly important. These newly arrived cells were found to proliferate *in situ* in crown-like structures^18^. Unfortunately, we did not measure proliferation in our samples. However, based on our findings and current literature, we believe it is likely that both recruitment and local proliferation take part in human AT immune cell accumulation.

Integrins are involved in trafficking^11^, adhesion and transmigration of monocytes^12^ and expression of the integrin CD11b on total monocytes was shown to be associated with CD68 gene expression in scAT biopsies^19^. However, our data did not reveal any association between integrin expression by separate monocyte subsets and macrophage subsets in vAT. These results thus argue against a need for CD11b and CD11c upregulation on monocytes for their recruitment to AT.

CX3CR1, which acts as a receptor for fractalkine (CX3CL1), has been shown to function as a chemoattractant and as an adhesion molecule^20^. In humans CX3CR1 has been implicated in obesity and type 2 diabetes^21^. However, there is conflicting data whether CX3CR1 is directly involved in monocyte recruitment to AT, AT inflammation and insulin resistance^22^,^23^. Devevre *et al*. showed that CX3CR1 expression was increased in all three monocyte subsets of obese individuals^24^. Our data showed increased CX3CR1 expression only on nonclassical monocytes although intermediate monocytes tended to show increased surface expression. Intriguingly, although intermediate monocytes were not associated with AT macrophages, we did found a positive association between CX3CR1 surface expression on these cells with CD11c^+^ macrophages in vAT. Oppositely, classical monocytes did not show enhanced CX3CR1 surface expression, suggesting that CX3CR1 does not affect the relationship between classical monocytes and CD11c^+^ macrophages in vAT.

Of interest is our observation that next to circulating monocytes and vAT macrophages, also circulating and vAT NK-cells were higher in obese individuals. Recent observations in mice show that NK-cells infiltrate vAT early during obesity development and contribute to the inflammatory environment causing insulin resistance^25^,^26^. It has been shown that blood NK-cells are increased obese individuals but displayed decreased function^27^. However, we did not observe an association between blood and vAT NK cells. Further studies are thus needed to clarify the potential role of NK-cells in obese humans.

Our results did not reveal associations between vAT or scAT T-cell subsets and circulating T-cells. In contrast with these data, McLaughlin *et al*. reported significant correlations between CD4^+^ Th cells and CD8^+^ Tc cells in blood and scAT in humans^28^. McLaughlin *et al*. presented T-cell subsets as % of total T cells while our data are presented relative to all circulating leukocytes or stromal vascular cells. While our data reflect a poor quantitative association between circulating and AT T-cell subsets, representing T-cell subsets as a fraction of total T-cells by McLaughlin *et al*. may reflect qualitative associations between blood and AT T-cell subsets. Thus, our data suggests that the predictive value of measuring circulating lymphocytes in blood as a reflection for immune cell infiltration in vAT is limited.

Finally, we analysed the association between immune cells in the two major AT depots. Since vAT is not readily accessible for research purposes, many studies use scAT biopsies as a marker for AT inflammation. Our data show that scAT is a proxy for determining M1 macrophages and CD4^+^ T-helper cells in human vAT. However, it should be kept in mind that inflammation is less pronounced in scAT than in vAT. In addition, our results show that this association is stronger in obese individuals, implicating that using scAT CD11c^+^ macrophages as a marker for vAT inflammation should be done cautiously. Intriguingly, the association between vAT and scAT CD4^+^ T-helper cells depended completely on lean individuals as this relation was no longer present in obese men. It is known that obesity leads to a shift in the balance of Th2 and Th1 cells^29^. Since we did not include intracellular markers of T-helper cell polarization, it is possible that preferential recruitment of certain T-cell subsets negatively affected these associations in obese individuals.

Our study has some limitations. First, only male subjects were included. Therefore, future studies are needed to investigate whether current findings are also true for female individuals. Second, next to M1 adipose tissue macrophages, CD11c is considered to be a marker of dendritic cells (DCs). Since most human DCs either express no CD11b or low levels of CD11b^30^ and since monocytes upregulate CD11b expression during their maturation into macrophages^31^, we identified adipose tissue macrophages as cells that express high levels of CD11b (see supplemental Fig. 1 for gating strategy). Comparing CD11c^+^ CD11b^+^ adipose tissue macrophages with CD11b^low/−^CD11c^+^ cells, reflecting DCs, we indeed found that CD11b^low/−^CD11c^+^ cells displayed higher expression levels of the DC markers CD141 and CD303 (suppl. Fig. 1c). Third, using biopsies from surgical procedures bears a risk of blood contamination of the tissue samples. Hence, part of the immune cells measured in these samples may be arising from residual blood. However, our data do not show any associations between the relative amount of T-cells, B-cells or NK cells between the circulation and adipose tissue, despite being identified by the same markers in blood and tissues. Therefore, we believe that the effects of blood contamination of the tissues used in this study are only minor. Finally, we did not correct our analyses for multiple testing. We acknowledge that this comprehensive evaluation of relations between different immune cell depots entailed multiple statistical tests, which may have increased the chance of false positive findings. On the other hand, rigorous adjustment for multiple testing would increase the chance that a real biological association is not detected. In this study, we explored hypotheses that were generated from basic science in order to provide a first line of human evidence as basis for further research. In the light of this aim, we argued that it would be undesirable to miss an association that does exist in the population. Therefore, and in agreement with current recommendations for observational studies, we did not formally adjust for multiple testing^32^.

In conclusion, our data show, independently from surface integrin expression, that blood classical monocytes are associated with CD11c^+^ macrophages in vAT.

## Additional Information

**How to cite this article**: Wouters, K. *et al*. Circulating classical monocytes are associated with CD11c^+^ macrophages in human visceral adipose tissue. *Sci. Rep.* **7**, 42665; doi: 10.1038/srep42665 (2017).

**Publisher's note:** Springer Nature remains neutral with regard to jurisdictional claims in published maps and institutional affiliations.

## Supplementary Material

Supplementary Information

## Figures and Tables

**Table 1:** Baseline parameters.

	Lean Subjects	Obese Subjects	P-value
Age (years)	51.5 (47.5–58.5)	49.0 (45.0–54.0)	0.210
BMI (kg/m^2^)	23.7 (22.7–24.8)	37.4 (36.1–39.4)	<0.001
Waist-to-hip ratio	0.98 (0.96–0.99)	1.09 (1.06–1.11)	<0.001
Plasma glucose (mM)	5.6 (5.2–5.9)	5.8 (5.5–7.2)	0.014
Serum Insulin (mU/L)	6.7 (5.5–12.1)	19.0 (15.0–29.5)	<0.001
HOMA-IR	1.71 (1.3–3.2)	5.7 (3.6–8.3)	<0.001
HbA1C (%)	5.2 (5.1–5.5)	6.0 (5.4–6.8)	<0.001
Fat percentage (%)	22.2 (19.8–27.9)	36.2 (34.6–38.3)	<0.001

Table shows baseline parameters from lean and obese individuals included in this study. Data are presented as median (interquartile range). Differences between lean and obese individuals are analysed with an independent student t-test. *P-value < 0.05 is considered statistically significant.

**Table 2:** Immune cell populations in blood.

	Lean Subjects	Obese Subjects	P-value
WBC (×10 * 9/L)	6.9 (6.3–9.5)	6.6 (5.7–8.4)	0.197
Lymphocytes (×10 * 9/L)	1.9 (1.6–2.3)	1.8 (1.5–2.2)	0.136
Monocytes (×10 * 9/L)	0.5 (0.4–0.7)	0.5 (0.4–0.6)	0.416
Basophils (×10 * 9/L)	0.05 (0.04–0.07)	0.04 (0.02–0.05)	0.060
Eosinophils (×10 * 9/L)	0.22 (0.16–0.37)	0.15 (0.08–0.26)	0.065
Neutrophils (×10 * 9/L)	4.3 (3.0–6.0)	3.7 (3.2–5.4)	0.533
B-cell (% of total)	3.5 (2.0–5.0)	3.7 (2.7–4.7)	0.786
T-cell (% of total)	58.5 (55.2–62.8)	52.8 (46.4–60.3)	0.371
CD4^+^ (% of total)	13.5 (10.5–18.8)	16.1 (12.1–18.6)	0.304
CD8^+^ (% of total)	5.0 (2.9–10.2)	6.5 (4.0–11.4)	0.295
T-helper (% of total)	11.8 (9.5–16.7)	14.9 (11.0–16.9)	0.286
T-regulatory (% of total)	1.4 (1.0–1.8)	1.4 (0.9–1.8)	0.856
NK-cells (% of total)	2.9 (1.8–4.7)	4.9 (3.1–6.5)	0.016*
Classical Mono (% of total)	4.6 (3.8–5.7)	6.5 (4.6–7.5)	0.012*
CD11c (MFI) (×10^3^)	5.1 (4.1–5.9)	6.3 (4.8–7.4)	0.242
CD11b (MFI) (×10^3^)	20.5 (17.9–25.0)	27.8 (24.6–31.3)	0.008*
CX3CR1 (MFI) (×10^3^)	4.6 (3.8–5.5)	5.4 (4.1–6.6)	0.097
Intermediate Mono (% of total)	0.2 (0.2–0.3)	0.3 (0.2–0.5)	0.077
CD11c (MFI) (×10^3^)	17.4 (16.0–19.1)	17.4 (16.3–21.2)	0.803
CD11b (MFI) (×10^3^)	23.2 (20.7–30.6)	30.7 (26.8–35.7)	0.022*
CX3CR1 (MFI) (×10^3^)	12.7 (9.7–15.8)	14.2 (11.9–16.0)	0.153
Nonclassical Mono (% of total)	0.5 (0.4–0.8)	1.0 (0.7–1.6)	0.000*
CD11c (MFI) (×10^3^)	12.2 (9.8–13.5)	15.1 (12.8–16.8)	0.098
CD11b (MFI) (×10^3^)	5.3 (3.2–6.6)	6.5 (4.7–7.3)	0.066
CX3CR1 (MFI) (×10^3^)	10.7 (8.7–12.7)	12.0 (10.4–13.7)	0.043*

Blood cells were either measured by an automated clinical assay (**×**10^9^ cells/L) or with flow cytometry (% of total cells) in whole blood. Data are presented as median (interquartile range). Differences in immune cell populations were analysed with linear regression with adjustment for age. ^*^P-value < 0.05 is considered significant.

**Table 3:** Immune cell populations in scAT and vAT.

		Lean Subjects	Obese Subjects	P-value
**Subcutaneous**	CD45^+^ (% of total)	48.3 (36.6–54.1)	38.4 (30.2–50.6)	0.571
**Adipose Tissue**	CD11c^−^ CD11b^+^ (% of total)	3.8 (0.9–7.2)	2.6 (1.7–5.1)	0.873
	CD11c^+^ CD11b^+^ (% of total)	5.6 (2.3–9.5)	6.9 (4.2–11.1)	0.587
	NK-cells (% of total)	3.0 (2.2–4.0)	3.1 (2.4–4.9)	0.971
	T-cells (% of total)	24.4 (18.7–33.0)	24.7 (21.4–27.6)	0.971
	T-helper cells (% of total)	14.0 (9.5–20.6)	11.2 (8.9–14.8)	0.583
	Cytotoxic T-cells (% of total	7.6 (5.6–9.6)	8.7 (6.0–10.4)	0.375
	B-cells (% of total)	0.7 (0.4–1.0)	0.7 (0.5–1.5)	0.862
**Visceral**	CD45^+^ (% of total)	41.9 (25.6–47.5)	55.7 (50.6–61.6)	<0.001*
**Adipose Tissue**	CD11c^−^ CD11b^+^ (% of total)	4 (1.4–4.5)	5.0 (3.6–7.8)	0.066
	CD11c^+^ CD11b^+^ (% of total)	2.3 (1.5–3.5)	5.8 (2.3–8.2)	0.009*
	NK-cells (% of total)	2.7 (1.6–4.1)	3.8 (2.7–5.8)	0.029*
	T-cells (% of total)	27.3 (15.5–40,6)	40.8 (24.1–46.2)	0.107
	T-helper cells (% of total)	12.9 (9.8–25.1)	16.6 (12.4–25.2)	0.786
	Cytotoxic T-cells (% of total)	8.8 (6.1–16.5)	14.8 (6.7–21.8)	0.173
	B-cells (% of total)	0.5 (0.2–3.3)	1.4 (0.9–2.2)	0.055

Immune cells were measured with flow cytometry in SVF from scAT and vAT biopsies. Data (% of total cells) are presented as median (interquartile range). Differences in immune cell populations were analysed with linear regression with adjustment for age. ^*^P-value < 0.05 is considered significant.
