# Supplementary material for: Circulating classical monocytes are associated with CD11c+ macrophages in human visceral adipose tissue
Source: Sci Rep. 2017 Feb 15;7:42665. doi: 10.1038/srep42665 (PMC5309742; doi:10.1038/srep42665)
Supplement: Supplementary Information [file srep42665-s1.doc]

**Circulating classical monocytes are associated with CD11c^+^ macrophages in human visceral adipose tissue.**

Kristiaan Wouters ^1,2^*, Katrien Gaens ^1,2^, Mitchell Bijnen ^1,2^, Kenneth Verboven ^3,4^, Johan Jocken ^3^, Suzan Wetzels ^1,2^, Erwin Wijnands ^1,5^; Dominique Hansen ^4^, Marleen van Greevenbroek ^1,2^, Adriaan Duijvestijn ^1,2^, Erik A. L. Biessen ^1,5,6^, Ellen E. Blaak ^3^, Coen D.A. Stehouwer ^1,2^; Casper G. Schalkwijk ^1,2^

**Supplemental information**


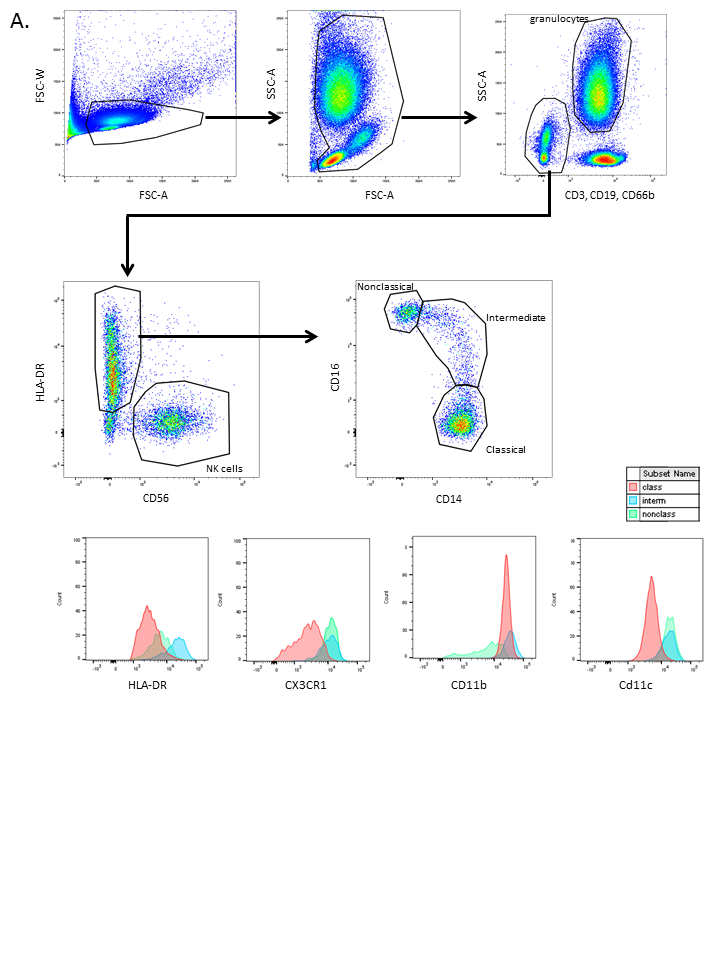


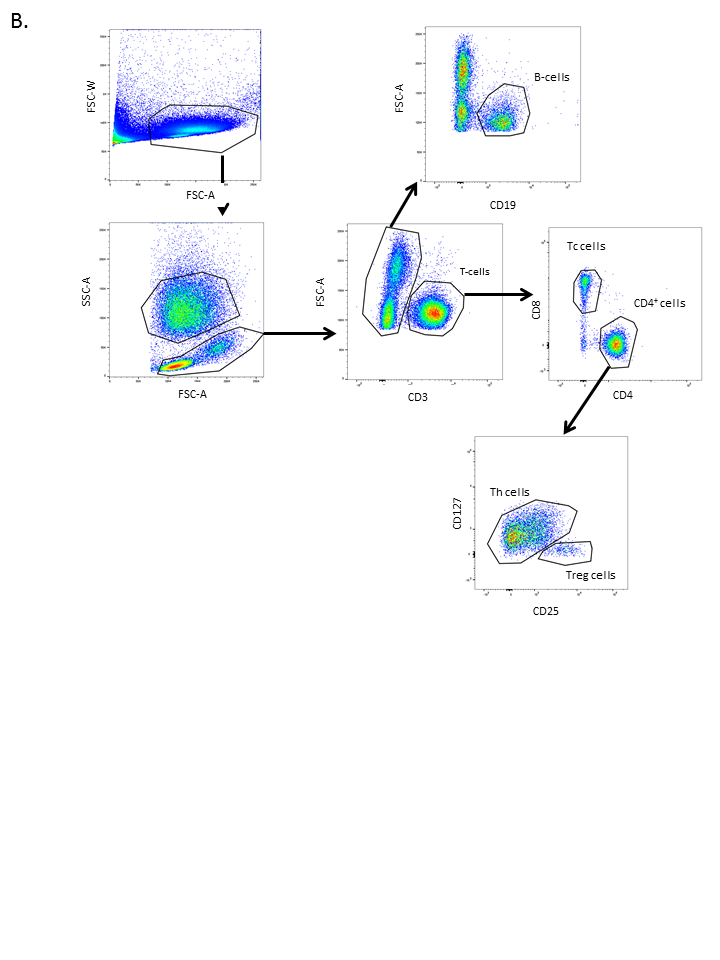


**
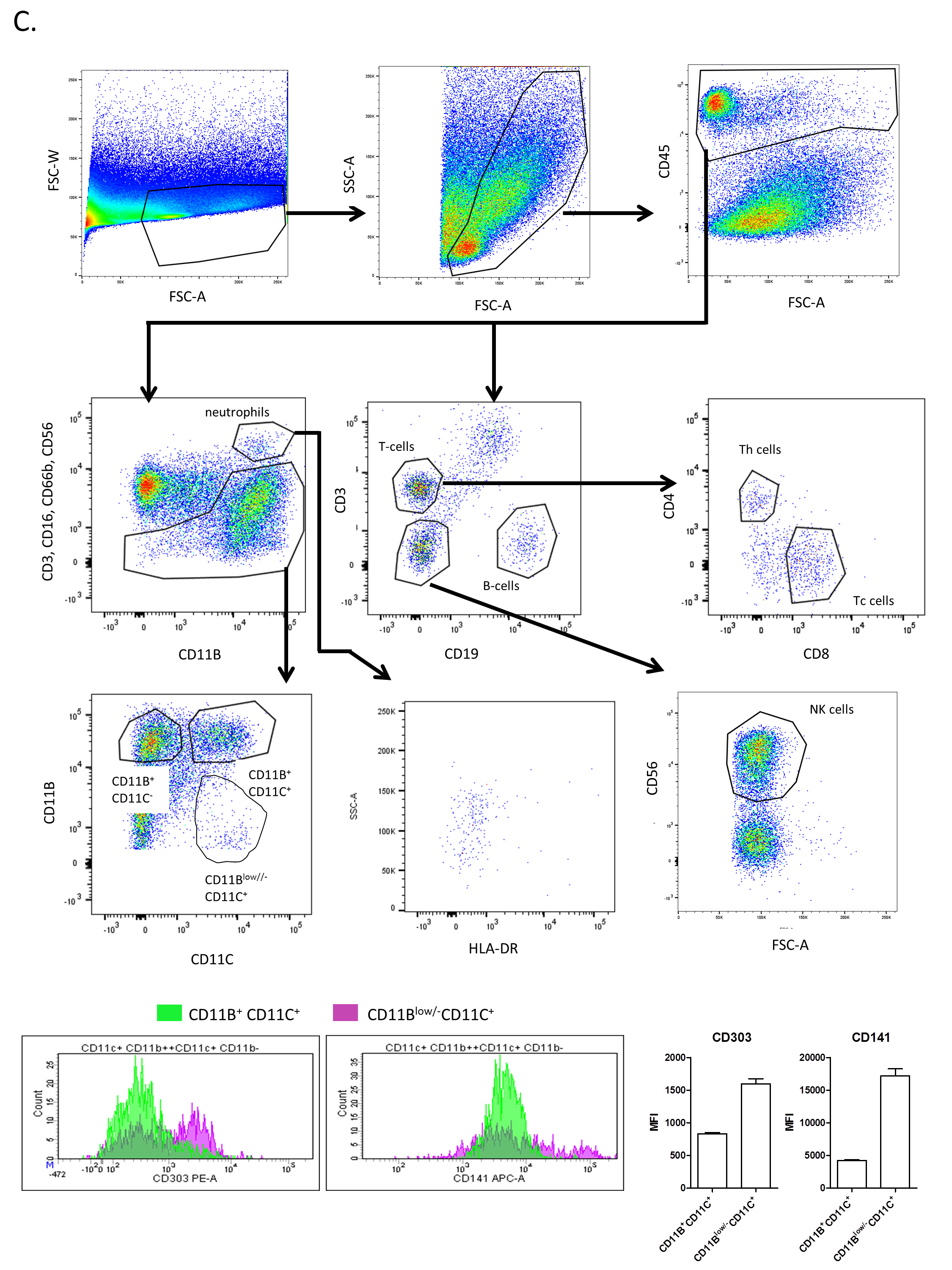
**

**Supplemental Fig. 1. Gating strategy**

**A.** From single blood cells, live cells were gated based on forward and side scatters. From live cells, negative (dump) selection was done based on CD3 (T cells), CD19 (B cells) and CD66b (granulocytes). Dump^-^ cells were gated for CD56 (NK cells) and HLA-DR (monocytes). HLA-DR^-^ cells were over 95% negative for CD14 and CD16 (data not shown). From monocytes, subsets were identified based on CD14 and CD16 expression. The identity of the different monocyte subsets was validated using surface expression of CD11C, HLA-DR and CX3CR1**.** The boundaries of gates to differentiate between classical, intermediate and nonclassical monocytes were defined for each participant based on the expression of these markers. Classical monocytes: CD11c^low^, CX3CR1^low^ and HLA-DR^low^; intermediate monocytes: CD11c^hi^, CX3CR1^int^ and HLA-DR^hi^; nonclassical monocytes: CD11c^hi^, CX3CR1^hi^ and HLA-DR^int^. **B.** From single blood cells, monocytes and lymphocytes were gated. B-cells were identified based on CD19 expression and T-cells by CD3. From total T cells, cytotoxic T cells were identified based on CD8 expression. From CD4-positive cells, Tregs (CD127^low^ CD25^high^) and T helper cells (CD127^high^ CD25^low^) were identified. **C.** SVF cells from vAT and scAT were gated for live single cells based on forward and side scatters. Immune cells were selected based on CD45 expression. Lymphocyte populations in AT were identified based on CD19 for B-cells and CD3 for T-cells. T-cells were further subdivided in CD8^+^ Tc cells and CD4^+^ Th cells. Non-B- and T-cells were gated for CD56 to identify NK cells. From CD45^+^ cells B-cells (CD19), T-cells (CD3), NK cells (CD56) and granulocytes (CD66b) were excluded for the identification of macrophages (defined as CD11B^hi^), which were subdivided in CD11B^+^CD11C^-^ and CD11B^+^CD11C^+^ macrophages. The expression of the dendritic cell markers CDCD303 and CD141 was analyzed confirming that CD11B^low/-^ cells express dendritic cell markers compared to CD11B^+^CD11C^+^ cells.
